# Effectiveness of health checkup with depression screening on depression treatment and outcomes in middle-aged and older adults: a target trial emulation study

**DOI:** 10.1016/j.lanwpc.2023.100978

**Published:** 2023-11-23

**Authors:** Yu-Ling Chen, Ming-Shiang Wu, Shih-Heng Wang, Yin-Ju Lien, Shih-Cheng Liao, Chia-Ming Chang, Wei-Lieh Huang, Chi-Shin Wu, Chih-Cheng Hsu

**Affiliations:** a National Center for Geriatrics and Welfare Research, National Health Research Institutes, Zhunan, Taiwan; b Department of Physical Education, National Taiwan University of Sport, Taichung, Taiwan; c Department of Occupational Safety and Health, College of Public Health, China Medical University, Taichung, Taiwan; d Department of Health Promotion and Health Education, National Taiwan Normal University, Taipei, Taiwan; e Department of Psychiatry, National Taiwan University Hospital, Hisn-Chu Branch, Hsinchu, Taiwan; f Department of Psychiatry, College of Medicine, National Taiwan University, Taipei, Taiwan; g Department of Psychiatry, Chang Gung Memorial Hospital at Linkou, Taoyuan, Taiwan; h Department of Psychiatry, National Taiwan University Hospital, Yunlin Branch, Yunlin, Taiwan; i Institute of Population Health Sciences, National Health Research Institutes, Zhunan, Taiwan

**Keywords:** Mass screening, Treatment outcome, Suicide, Hospitalization, Age

## Abstract

**Background:**

Adult preventive health checkups with depression screening were launched in August 2011 in Taiwan; however, its impact has not yet been evaluated. This study aimed to use real-world data to assess the effectiveness of depression screening among middle-aged and older adults.

**Methods:**

A total of 4,972,228 adults aged 40 years and above who participated in a health checkup with depression screening between 2013 and 2019 and the same number of unscreened counterparts were included. The target trial emulation study was conducted to estimate the hazard ratios (HRs) for newly treated depression, psychiatric hospitalisation, and suicide. The changes in HRs during the study period were assessed using interval Cox models.

**Findings:**

The screening group had a higher rate of newly treated depression (HR 1.63 [95% CI 1.62, 1.64]) and a lower risk of psychiatric hospitalisation (HR 0.93 [95% CI 0.91, 0.95]). There was a null association between depression screening and suicide; however, a higher suicide risk was found in screened older adults aged 65 years and above. Only 10.8% received depression treatment during the study period among the screen-positive individuals.

**Interpretation:**

Health checkups with depression screening could potentially promote depression treatment and reduce the risk of psychiatric hospitalisation; however, there was no effect on suicide. The treatment rate for depression remained low after screening for depression. Further attention to enhance referral and treatment is required.

**Funding:**

The study was funded by the 10.13039/501100004737National Health Research Institutes, Taiwan.

**Research in context**

**Evidence before this study**

Prior to conducting this study, we searched PubMed, Google Scholar, the international prospective register of systematic reviews (PROSPERO) records, and Cochrane Library for the existing evidence of randomized control studies, observational studies, and systematic reviews in November 2022 for studies in English, with the terms “screening for depression” and “depression screening”. Studies included in the published guidelines of the USA, Canada, and the UK for depression screening were also reviewed. We included various screening tools, encompassing online, face-to-face, and paper-based questionnaires. Our investigation revealed a disparity between the published guidelines and the limited evidence on the effect of depression at the randomised controlled trial (RCT) level. Some evidence generated from observational studies showed a positive effect on the diagnosis and treatment rate of depression screening, and only two studies reported its effect on mental health outcomes (i.e., improvement in depression symptom scores).

**Added value of this study**

To our knowledge, this is the first study to analyze the effectiveness of general health checkups with depression screenings. We applied real-world data from 4,972,228 adults undergoing depression screening and their non-screening-matched counterparts to the target trial emulation. Our main findings show that the screening group had an increasing rate of newly treated depression and a lower risk of psychiatric hospitalisation. However, there was no association between screening for depression and suicide. In addition, the treatment rate was still low, regardless of screening status.

**Implications of all the available evidence**

Our study supports the effectiveness of health checkups with depression screening to increase the identification and treatment of depression and reduce the risk of psychiatric hospitalisation. However, the effect size was small, which might be because the treatment rate remained low, even in screen-positive patients. Providing incentives for referral or enhancing depression care training in primary care practices may improve depression outcomes.

## Introduction

Globally, depressive disorders are among the most burdensome illnesses. Nevertheless, a substantial proportion of depression cases remain unrecognized and untreated; the treatment rate is only 34.8% globally, and it can be as low as 16.8% in low-income countries^1^ and 27% in middle-aged and older adults in Taiwan.^2^ Depression screening is a quick and straightforward approach to address underdiagnosis and undertreatment issues. Some, but not all, randomized controlled trials (RCTs) suggested that screening for depression might improve depression diagnosis or treatment rate.^3^^,^^4^ Therefore, the US Preventive Service Task Force recommends screening the adult population for depression if adequate support systems are available.^5^ However, there are debates, including the method and frequency of screening, on introducing screening for depression in primary care settings.^6^ Universal depression screening is not recommended in the UK and Canada due to insufficient evidence to support the effectiveness of universal depression screening.^7^^,^^8^

In order to inform the policy and to understand the direct effect of depression screening, Thombs et al. suggested that evidence should be generated from RCT studies that (i) conduct randomization prior to screening, (ii) exclude patients with existing depression, and (iii) apply similar management to screening and no-screening participants.^9^ Amongst the existing RCT studies, only one met these criteria to test the direct effect of depression screening among postpartum women,^4^ and most studies tended to focus on collaborating screening with integrating care systems or education programs for healthcare providers.^9^ The other fundamental limitation of the current evidence at the RCT level was a small sample size, and the results were lack of generalizability.^10^ Furthermore, only a few studies explored the effect of depression screening on health outcomes.^3^^,^^4^

In Taiwan, adults over 40 years of age are offered adult preventive healthcare checks covered by the Health Promotion Administration. Brief screening for depression has been included as part of the health checkup since August 2011; however, its effectiveness has not yet been evaluated. Although RCT is an ideal approach to understanding the effect of depression screening, it would be impossible, given that universal depression screening was initiated in Taiwan. Therefore, in this current study, we aimed to emulate a target trial to evaluate the effectiveness of depression screening using observational data.^11^

## Methods

### Data source

This emulating target trial study obtained data from the Adult Preventive Health Checkup (APHC) program from 2012 to 2019, which were linked to Taiwan's National Health Insurance (NHI) claims database and Taiwan's National Mortality Registry. The APHC was provided by Taiwan's Health Promotion Administration, a government organization responsible for health promotion and non-communicable disease prevention in Taiwan. APHC is a free preventive service generally offered once every three years and once a year to adults aged 40–64 years and 65 and above in Taiwan, respectively. The health checkup includes an essential physical examination (e.g., height, weight, vision, oral examination, blood pressure), blood tests (e.g., blood sugar, cholesterol, triglycerides), and urine tests (e.g., urine protein). Since August 2011, the APHC has included screening for depression, glomerular filtration rate, and high-density lipoprotein cholesterol levels.

The NHI claims database was derived from Taiwan's National Health Insurance Program, a universal compulsory program that includes approximately 99% of the 23 million Taiwanese population. The NHI claims database provided information about the insureds' clinical diagnoses and prescription records. The clinical diagnoses were coded based on the International Classification of Diseases (ICD)-9 before 2016 and updated to ICD-10 since 2016.

### Eligible criteria

To emulate the target clinical trial, we identified eligible subjects aged 40 years or older who had not yet received health checkups with depression screening. They should have a one-year washout period, 2012, to exclude those who had received a health checkup. In addition, eligible adults should be ever enrolled in the NHI program between Jan 1, 2013 and Dec 31, 2019. Subjects with missing data in birth year, sex, monthly income, or residential area were excluded. Furthermore, eligible subjects diagnosed with depressive disorders or bipolar spectrum disorder before treatment assignment were excluded.

### Treatment assignment

As part of the self-evaluating health questionnaire included in the health checkup, depression was screened using the Whooley questions, which is a two-question instrument: (1) “During the past month, have you often been bothered by feeling down, depressed, or hopeless?” and (2) “During the past month, have you often been bothered by little interest or pleasure in doing things?”. A response that answered yes to either of the questions was considered a positive test. This two-question instrument has a sensitivity of 89%–96% and a specificity of 51%–72% for diagnosing major depression.^12^ In the target trial, eligible individuals would be randomly assigned to either the screening or the no-screening group. In this emulation study, adults who underwent health checkups with depression screening were identified as the screening group. According to the sequence of the screening date of each case, an age- and sex-matched individual who had not yet undergone a health checkup was randomly selected from the abovementioned eligible subjects as an unscreened subject. An unscreened subject could be repeatedly selected or become a screened one at a later time during the study periods. Given that randomization assignments were impossible in observational studies, the different characteristics of screened and unscreened subjects would confound our results. Therefore, based on baseline characteristics, propensity score weighting was used to account for the differences between the screened and unscreened groups.

These characteristics included residential area, monthly income, Charlson comorbidity index (CCI), number of outpatient visits in the preceding year, and common comorbid medical and psychiatric disorders. The follow-up began at time zero, defined as “time participating in the health checkup” for the screened individuals and the same date for their matched counterparts. Individuals were followed up until the study outcomes, death, or end-of-follow-up (i.e., Dec 31 2019), whichever occurred first. Supplementary Table S1 presents the protocol of the target trial and the emulation procedure for the target trial.

### Outcomes

Newly treated depression was identified from outpatient claims records based on ICD codes, including major depressive disorder (ICD-9-CM: 296.2, 296.3; ICD-10: F32.0–F32.9, F33.0–F33.9), minor depression, including dysthymia (ICD-9-CM: 300.4; ICD-10-CM: F34.1), and depressive disorder not otherwise specified (NOS; ICD-9-CM: 311). Psychiatric hospitalization was considered an adverse outcome if patients did not receive adequate treatment. It's important to note that psychiatric hospitalizations encompassed a broader range of psychiatric disorders, not restricted solely to depression. This approach acknowledged the possibility that patients with untreated depressive symptoms might result in other severe psychiatric conditions, such as substance use or suicidal attempts. Suicide was another outcome identified from the cause-of-death code (ICD-9 code: E950–959; ICD-10 code: X60–X84 or X87.0) in Taiwan's National Mortality Registry. The cause of all unnatural deaths, including suicide, homicide, or accidental death, is determined by a prosecutor. The accuracy of suicide statistics was confirmed through a psychological autopsy study conducted in the early 1990s, which found that only 2 out of 117 suicides were incorrectly classified as accidental deaths.^13^

### Statistical analysis

The descriptive statistics of the baseline characteristics of the screening and no-screening groups are reported in Supplementary Table S2. The 0.1 threshold of the standardized mean difference was used to assess whether the balance was achieved by propensity score weighting.

We conducted a per-protocol analysis to estimate the effect of health checkups with depression screening on the study outcomes. All participants were followed until the study outcome, deviation from their assigned strategy, or the end of the study period (Dec 31 2019), whichever occurred first. Deviation from their assigned strategy occurred if the unscreened subjects underwent health checkups with depression screening during the follow-up period. The cumulative incidence of the study outcomes during the follow-up period was plotted. Death was considered a competing risk in our analysis. Therefore, we conducted a competing risk survival analysis^14^ to estimate the hazard ratio (HR) and 95% confidence interval (CI) for the study outcomes. We conducted a sensitivity analysis using intention-to-treat analysis, the same as the per-protocol analysis, except that the subjects were not censored for deviation from the assigned group. In addition, we conducted another sensitivity analysis using per-protocol analysis with unweighted data and multivariate Cox regression models.

Subgroup analyses were carried out by patient age groups (40–54, 55–64, and ≥65 years), sex, and urbanicity (urban, suburban, and rural areas) to evaluate the modifying effect on the association between depression screening results and study outcomes. Propensity score weighting was conducted for each subgroup to make sure the baseline characteristics were balanced in subgroup analyses.

Given that the proportional assumption might be violated, applying the standard Cox proportional hazard regression model would generate an average risk, which might lead to misleading results. Therefore, we used an interval Cox proportional hazard model to explore the change in HR during the study periods.

We also conducted a post hoc analysis to explore depression outcomes among patients with newly treated depression during the follow-up period. Based on screening status, patients were categorized into no-screening, screen-positive, who had any positive answer to Wooley's questionnaire, and screen-negative. Patient characteristics, including age at diagnosis, were assessed at the time depression treatment was initiated. The crude and adjusted HRs of screening status for psychiatric hospitalization and suicide were estimated using multivariate Cox regression models.

### Statement of ethics

This study was approved by the Research Ethics Committee of the National Health Research Institutes (EC1101103-E). The informed consent is waived due to personal information being encrypted in the NHI claims database.

### Role of the funding source

The funders had no role in study design, data collection, data analysis, interpretation, writing of the report.

## Results

A total of 4,972,228 screened individuals and an equal number of comparisons were made (Supplementary Figure S1). The average follow-up period was 4.1 years for screened individuals and 3.2 years for unscreened subjects because 1,089,243 of them attended health checkups and became screened groups during the follow-up periods. This study included a higher proportion of individuals aged 40–54 years (47%), and 55% were females. Generally, the screened individuals were more likely to live in a suburban or rural area, have a monthly income between NT$20001–40000 (considered middle-class income), and have a higher number of outpatient visits than the unscreened subjects (Supplementary Table S2). After weighting, the baseline characteristics of the two groups were balanced; the standardized mean difference of all variables was <0.1.

### Rate of newly treated depression

**Supplementary Figure S2:**
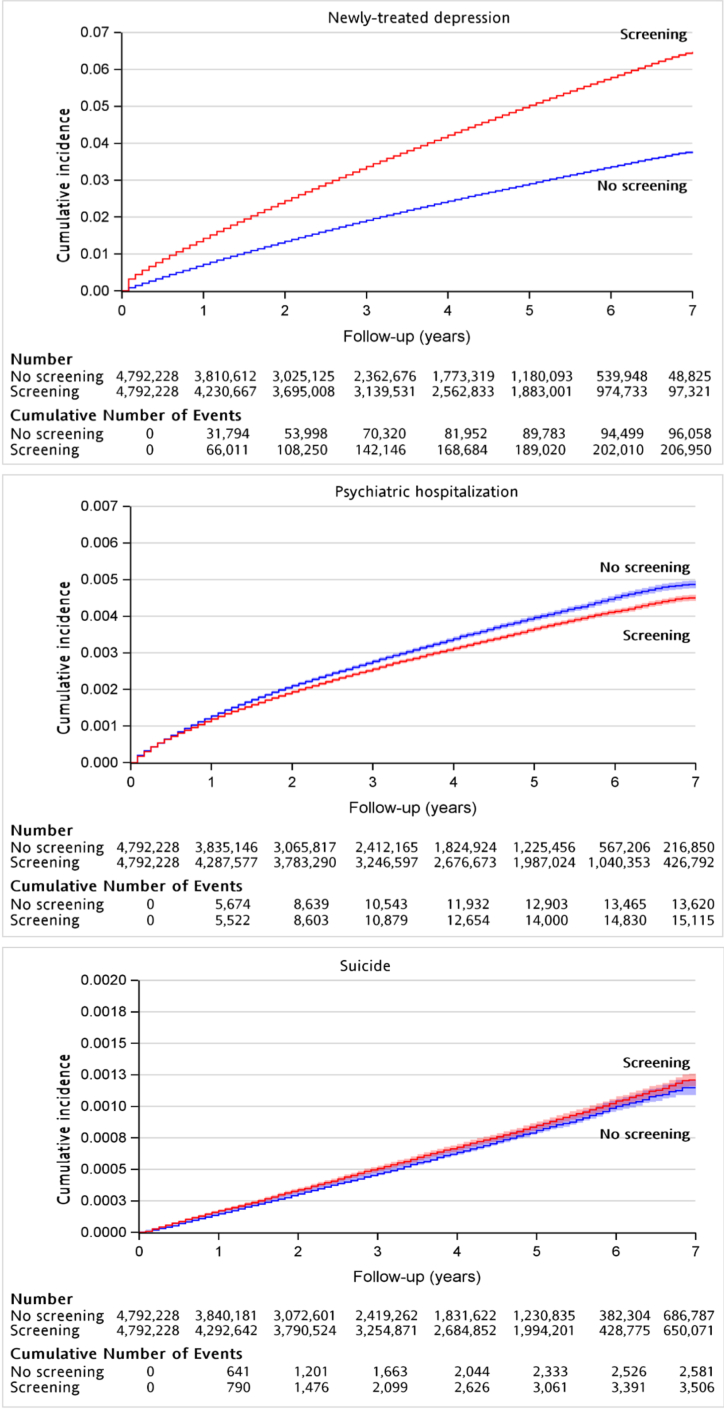


After propensity score weighting, there were 200,817 and 99,531 patients with newly-treated depression in the screened and unscreened groups, respectively. The incidence per 1000 person-years was 10.48 for the screened group and 6.61 for the unscreened group. It was found that those who underwent a health checkup with depression screening had a higher likelihood of newly treated depression (competing HRs = 1.66; 95% CI [1.63, 1.66]; p < 0.001). Cumulative incidence plots for the study outcomes are shown in Supplementary Figure S2. This trend was observed across all groups among the subgroups (Table 1). The HRs were lower among those aged 65 or more, females, and individuals who lived in urban areas; however, the differences were mild, ranging from 1.53 to 1.65.

**Table 1:** Rate of newly treated depression among patients with and without depression screening using propensity score weighting.

	Depression screening	No. of persons	No. of 1000 person-years	Rate per 1000 person-years	No screening	No. of persons	No. of 1000 person-years	Rate per 1000 person-years	Hazard ratios (95% CI)	p value
	No. of events				No. of events					
**Overall**	200,817	4,972,228	19,155	10.48	99,531	4,972,228	15,048	6.61	1.65 (1.63, 1.66)	<0.001
**Age groups**										
40–54	82,199	2,241,025	8664	9.49	41,277	2,210,131	7115	5.80	1.65 (1.64, 1.67)	<0.001
55–64	60,842	1,416,217	6001	10.14	28,723	1,446,745	4538	6.33	1.64 (1.62, 1.67)	<0.001
≥65	57,545	1,134,986	4489	12.82	29,663	1,135,352	3392	8.75	1.53 (1.51, 1.55)	<0.001
**Sex**										
Male	70,134	2,156,503	8442	8.31	35,369	2,155,544	6866	5.15	1.64 (1.62, 1.66)	<0.001
Female	130,666	2,635,725	10,712	12.20	64,166	2,636,684	8181	7.84	1.60 (1.58, 1.61)	<0.001
**Urbanicity**										
Urban	96,102	2,469,914	9027	10.65	56,953	2,469,682	8433	6.75	1.60 (1.59, 1.62)	<0.001
Suburban	77,864	1,742,933	7519	10.36	32,789	1,743,108	5075	6.46	1.65 (1.63, 1.67)	<0.001
Rural	27,007	579,380	2641	10.22	10,112	579,438	1560	6.48	1.63 (1.59, 1.67)	<0.001

### Risk of psychiatric hospitalization

Table 2 presents that there were 15,157 patients with psychiatric hospitalization in the screened group, while the unscreened group had 13,417 such patients. The incidence was 0.77 per 1000 person-years for the screened group and 0.88 for the unscreened group. The screened individuals had less risk of psychiatric hospitalization compared to their comparisons (competing HRs = 0.93; 95% CI [0.91, 0.95]); p < 0.001). Further subgroup analyses showed this trend was significant in those aged 55–64 years, who lived in the suburban or rural areas, and females.

**Table 2:** Risk of psychiatric hospitalization among patients with and without adult health checkups and depression screening using propensity score weighting.

	Depression screening	No. of persons	No. of 1000 person-years	Rate per 1000 person-years	No screening	No. of persons	No. of 1000 person-years	Rate per 1000 person-years	Hazard ratios (95% CI)	p value
	No. of events				No. of events					
**Overall**	15,157	4,972,228	19,699	0.77	13,417	4,972,228	15,307	0.88	0.93 (0.91, 0.95)	<0.001
**Age groups**										
40–54	7613	2,241,025	8884	0.86	6722	2,210,131	7218	0.93	0.98 (0.95, 1.01)	0.175
55–64	4063	1,416,217	6172	0.66	4026	1,446,745	4617	0.87	0.81 (0.78, 0.85)	<0.001
≥65	3416	1,134,986	4641	0.74	2714	1,135,352	3469	0.78	1.01 (0.96, 1.06)	0.678
**Sex**										
Male	7983	2,156,503	8616	0.93	6811	2,155,544	6947	0.98	1.01 (0.98, 1.04)	0.552
Female	7165	2,635,725	11,082	0.65	6641	2,636,684	8360	0.79	0.87 (0.84, 0.90)	<0.001
**Urbanicity**										
Urban	6867	2,469,914	9285	0.74	6894	2,469,682	8582	0.80	0.97 (0.94, 1.00)	0.071
Suburban	5833	1,742,933	7733	0.75	4531	1,743,108	5162	0.88	0.93 (0.89, 0.97)	<0.001
Rural	2548	579,380	2712	0.94	1884	579,438	1586	1.19	0.88 (0.82, 0.93)	<0.001

### Risk of deaths by suicide

In the screened group, there were 3414 patients with death by suicide, compared to 2616 in the unscreened group. The incidence rate for both groups was 0.17 per 1000 person-years. Overall, the hazard ratios for depression screening for suicide were not statistically significant (Table 3). However, in subgroup analyses, we found that depression screening was associated with a higher suicide risk among adults aged ≥65 years (competing HRs = 1.15; 95% CI [1.05, 1.25]; p = 0.002).

**Table 3:** Risk of suicide among patients with and without adult health checkups and depression screening using propensity score weighting.

	Depression screening	No. of persons	No. of 1000 person-years	Rate per 1000 person-years	No screening	No. of persons	No. of 1000 person-years	Rate per 1000 person-years	Hazard ratios (95% CI)	p value
	No. of events				No. of events					
**Overall**	3414	4,972,228	19,741	0.17	2616	4,972,228	15,342	0.17	1.01 (0.96, 1.06)	0.720
**Age groups**										
40–54	1153	2,241,025	8906	0.13	994	2,210,131	7237	0.14	0.93 (0.85, 1.01)	0.084
55–64	982	1,416,217	6183	0.16	765	1,446,745	4627	0.17	0.96 (0.87, 1.05)	0.371
≥65	1276	1,134,986	4650	0.27	855	1,135,352	3476	0.25	1.15 (1.05, 1.25)	0.002
**Sex**										
Male	2319	2,156,503	8638	0.27	1819	2,155,544	6964	0.26	1.03 (0.97, 1.10)	0.331
Female	1101	2,635,725	11,102	0.10	789	2,636,684	8377	0.09	1.04 (0.95, 1.14)	0.439
**Urbanicity**										
Urban	1370	2,469,914	9305	0.15	1321	2,469,682	8599	0.15	0.95 (0.88, 1.03)	0.230
Suburban	1437	1,742,933	7750	0.19	910	1,743,108	5174	0.18	1.06 (0.97, 1.15)	0.180
Rural	651	579,380	2719	0.24	368	579,438	1590	0.23	1.03 (0.91, 1.17)	0.642

### Sensitivity analyses

The results of the intention-to-treat analysis were consistent with the results of the per-protocol analysis; however, the magnitude of the associations was generally smaller. Notably, the suicide risk from depression screening among those aged 65 years was not statistically significant (Supplementary Table S3). The results based on unweighted data with multivariate Cox regression model were also consistent with those of per-protocol analysis (Supplementary Table S4).

### Interval cox model

**Fig. 1:**
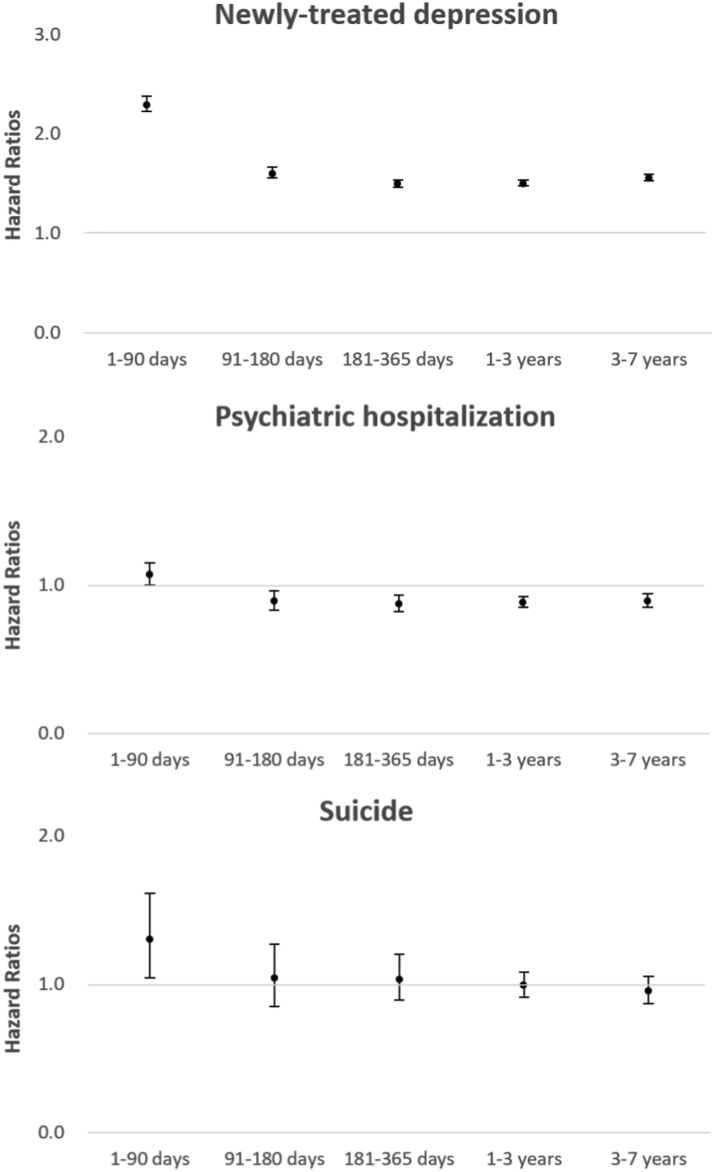
**Adjusted hazard ratios for newly treated depression, psychiatric hospitalization, and suicide from per-protocol analyses using interval Cox regression models**.

Over the 7-year follow-up period, interval analyses showed that HRs changed over the study period. The rate of newly treated depression increased and persisted throughout the study period. The hazard ratios of depression screening for psychiatric hospitalization were elevated in the first 90 days after screening (competing HR = 1.07; 95% CI [1.00, 1.15]; p = 0.039). However, since then, the screening group had a lower likelihood of psychiatric hospitalization compared to the no-screening group shown in Fig. 1. Furthermore, we found that the screening group had a higher risk of suicide within the first 90 days after the health checkup (competing HR 1.30 [95% CI 1.04, 1.61]; p = 0.021) compared to the control group. However, this difference was not significant. The details of the results obtained using the interval Cox regression model are shown in Supplementary Table S5.

### Post hoc analysis for patients with newly treated depression

Based on unweighted raw data, 125,213 individuals (2.6% of the screening groups) screened positive. During the follow-up period, 13,559 (10.8%), 193,392 (4.0%), and 96,058 (1.9%) patients were newly treated for depressive disorders in the screen-positive, screen-negative, and no-screening groups, respectively. Supplementary Table S6 shows that the mean age at treatment initiation for the screen-positive group was younger (mean age ± SD 58.7 ± 11.7 years) than that for the screen-negative (60.5 ± 11.9 years) and no-screening groups (60.8 ± 12.9 years). Compared to the no-screening group, the screen-negative individuals had a lower risk of psychiatric hospitalization; however, the screen-positive patients did not have a significantly lower risk (Supplementary Table S7). There was also no significant difference in the risk of suicide across these groups. The percentage of psychiatric hospitalization and suicide among patients treated for depression is shown in Supplementary Figure S3.

## Discussion

This emulating target trial study found that depression screening was associated with an higher rate of newly treated depression and lower risks for psychiatric hospitalization; however, there was no association with suicide over the study period. Using the interval Cox model, we found that the risk of depression screening for psychiatric hospitalization and suicide was higher in the first 90 days after depression screening, but this risk decreased afterward, and they had a lower risk for psychiatric hospitalization and an insignificant risk for suicide during the following period. Post hoc analysis showed that depression screening promotes early treatment; however, the treatment rate remains low. Only 10.8% of the screen-positive individuals were treated during the study period.

### Comparison with other studies

Evidence from clinical trials which investigated the effect of depression screening is limited.^10^ Three trials showed that depression screening in primary care settings had little impact on the diagnosis or treatment rate.^3^^,^^15^^,^^16^ However, one study revealed that depression screening for postpartum women had a higher diagnosis and treatment rate.^4^ In observational studies, most of them demonstrated an increase in the diagnosis of depression after screening in perinatal women,^17^^,^^18^ patients with cardiometabolic disorders,^19^ and adults in primary care settings.^20^

Nevertheless, the effect of depression screening on depression outcomes remains unclear. Most trials for middle-aged or older adults in primary settings did not demonstrate the benefits of screening for depression outcomes^3^^,^^15^^,^^16^; the null findings might be due to the limited sample size (n <300) for each arm. To the best of our knowledge, no observational studies have explored the effect of depression screening on health outcomes, such as psychiatric hospitalization or suicide.^17^, ^18^, ^19^, ^20^

### Depression treatment

We found that depression screening was associated with a higher rate of newly treated depression, which persisted throughout the study period. In the post hoc analysis of those with newly treated depression, we found that those who were screen-positive were younger than those who were unscreened. This finding indicates that screening for positive results could promote early treatment for depression. Even those with screen-negative results had earlier treatment than no-screening subjects. Given that we only assessed the results of the first depression screening, those with negative screen results initially might repeatedly attend health checkups, be screen-positive, and be treated in the following period. In addition, the education or consultation for individuals who received the health checkup might deliver health information, which could lead to early diagnosis and treatment. Furthermore, individuals who attended health checkups might use health services more frequently and have higher opportunities to receive psychiatric evaluations. In terms of subgroup analyses, we found that the treatment rate increased with age, female sex, and urban area in both groups, which might be explained by the depression stigma that was higher among men, the younger generation, and rural residents.^21^ Notably, the HRs for newly treated depression were slightly higher among those aged 40–64, males, and individuals who lived in rural or suburban areas. These findings indicate that depression screening could be useful, especially for those at risk of underdiagnosis and undertreatment.

### Psychiatric hospitalisation

The current study showed that the risk of psychiatric hospitalisation was higher shortly after screening but declined after 90 days. The transient increases in hospitalization could be explained by individuals with severe symptoms being recommended for psychiatric hospitalization shortly after referral and clinical assessment.^3^ Except for the first 90 days, the lower risk of psychiatric hospitalization indicated that depression screening could promote earlier treatment and potentially prevent the depression from worsening during the follow-up period.

Through subgroup analyses, we found that the screened individuals in the 55–64 age group, females, and those living in suburban or rural areas were associated with a significantly lower risk of psychiatric hospitalization compared to their unscreened counterparts. The underlying mechanism for the modifying effects was not clear; however, we thought that the treatment rate, adherence, and responses determined the effectiveness of depression screening on health outcomes. Previous studies showed that antidepressant adherence was relatively poor at a young age.^22^ Nonetheless, older adults with cognitive impairment had a poor response.^23^ These factors might explain our finding of the modification effect of age. In addition, females also had better treatment adherence in middle-aged and older adults.^22^ Mental health stigma also affected help-seeking behaviour, which was stronger in males than females.^24^ Therefore, there was no significant benefit in reducing psychiatric hospitalization among men. Although individuals living in rural or suburban areas have relatively insufficient healthcare resources, they may have stronger motivations for treatment and better responses than those in urban areas.^2^

### Suicide

Overall, we did not find a significant association between depression screening and deaths by suicide. The null findings might be due to the multifactorial causes of suicide. Suicide cases may potentially arise from psychiatric disorders other than depression, even in the absence of a formal psychiatric diagnosis.^25^ In addition, the screening questions were primarily aimed at identifying depression rather than suicide risk. The limited sensitivity of the questions for detecting suicide risk and the low treatment rate among those who screened positive could have contributed to this lack of association. Nevertheless, individuals who were severely depressed and screened positive likely received treatment for depression, suggesting that the screening process might still potentially help prevent suicide.

Of note, we found that adults over 65 years who attended health checkups with depression screening had a higher risk of suicide compared to those who did not. The Interval Cox model found that suicide risk was elevated immediately after a health checkup. The transient increase in suicide risk might be due to newly diagnosed severe diseases or health conditions after a health checkup,^26^ especially in older adults. Although we believe that the potential adverse effect of depression screening among older adults is less likely, this issue warrants further investigation.

### Low treatment rate

We also found that treatment rates were low. Even for screen-positive patients, the treatment rate was only 10.8% during the study period. This finding is in line with our previous study, which showed the treatment rate in Taiwan was lower than the global average.^2^ The low treatment rate also partially explained the effect size of depression screening was small for psychiatric hospitalization. Depression screening primarily took place as a self-administered health survey during the checkup. After completing the checkup, patients were offered a face-to-face consultation session. The primary care physician would provide health education, treatment plans, referrals, or observation recommendations based on the health survey results. However, there can be variations across primary care physicians due to the lack of standardized follow-up care for screened-positive individuals. Providing incentives for a referral or enhancing depression care training in primary care practice may improve depression outcomes.^27^

### Limitations

Our study has several limitations. First, although a target trial was emulated, this study is observational in nature. Potential unmeasured factors, such as socioeconomic status, patients' health behaviours, educational level, and healthcare accessibility, might have confounded our results. A pragmatic randomized clinical trial is necessary to confirm the effect of depression screening. Second, depression screening was conducted during the health checkup. Thus, our findings were attributed not only to depression screening but also to the impact of health checkups on other common chronic diseases. Third, Whooley questions are not validated in Taiwan, and their sensitivity and specificity are unclear. Further research is needed to evaluate the instrument's effectiveness and applicability locally. Fourth, this study did not directly measure patients' well-being; instead, we relied on treatment and admission as proxies. Fifth, some individuals may seek self-pay psychotherapy without a formal diagnosis or referral, which would not be recorded in the claims database. Although we assumed that the percentage of such cases is relatively low, we could not locate specific statistics to quantify this proportion. Sixth, this study was exploratory in nature and did not apply corrections for multiple comparisons in statistical analysis, which may increase Type I error rates. Finally, we only assessed the effect of the first health checkup on screening for depression. The participants might have repeatedly attended the health checkup; thus, the overall effect might also be due to the repeated checkup rather than only the first checkup.

### Implications

Incorporating depression screening into routine health checkups may aid in identifying and treating individuals with depression while potentially reducing psychiatric hospitalization. This finding supports preventive care that includes mental health assessments. However, the study reveals a persistently low treatment rate for depression, indicating the need for improved coordination between primary care providers and mental health specialists. Strategies addressing barriers to treatment, such as stigma, accessibility, and awareness, should be developed. Ensuring appropriate follow-up care and treatment for individuals identified through screening requires intensified efforts. Healthcare providers should facilitate timely referrals and access to mental health services for individuals screening positive for depression. Although depression screening did not demonstrate a significant association with suicide in the overall population, it underscores the importance of targeted suicide prevention strategies tailored to older adults, considering their unique vulnerabilities and risk factors. This study offers valuable insights to guide clinical practitioners and public health policymakers in optimizing depression screening programs, enhancing the referral and treatment process, and prioritizing treatment rates. Further cost-effectiveness analysis and evaluation of referral pathways are needed to improve depression screening strategies.

### Conclusion

This study used data from a nationally representative sample to emulate a target trial, and we found that health checkups with depression screening could increase depression treatment and reduce the risk of psychiatric hospitalization. However, we did not find an association between depression screening and deaths by suicide in the study population. The treatment rate was still low among screening-positive patients. It is necessary to optimize depression screening programs, improve referral and treatment procedures, and prioritize treatment rates. Notably, this emulation study is observational in nature, which was affected by confounding factors; a pragmatic randomized clinical trial is required to confirm the effects of depression screening.

## Contributors

All authors contributed to the study concept and design, analysis and interpretation of data, and critical revision of the manuscript for important intellectual content. CSW acquired the data and obtained funding. YLC, CSW drafted the manuscript. MSW, CSW did the statistical analysis. CCH is the guarantor. All the authors refined the various versions of the full paper and approved the final manuscript. The corresponding author attests that all listed authors meet authorship criteria and that no others meeting the criteria have been omitted.

## Data sharing statement

Data collected for this study are proprietary of the Health and Welfare Data Science Center at the Ministry of Health and Welfare, which was only available to granted researchers with permission.

## Editor note

The Lancet Group takes a neutral position with respect to territorial claims in published maps and institutional affiliations.

## Declaration of interests

During preparing this work, the authors used ChatGPT-3.5 and Grammarly to enhance writing quality and rectify grammar errors. Subsequently, the authors reviewed and edited the content, assuming full responsibility for the publication's integrity.

The authors declared no relevant conflict of interest.

## References

[1] Mekonen T., Chan G.C.K., Connor J.P., Hides L., Leung J. (2021). Estimating the global treatment rates for depression: a systematic review and meta-analysis. J Affect Disord.

[2] Chang T.-Y., Liao S.-C., Chang C.-M. (2022). Barriers to depression care among middle-aged and older adults in Taiwan's universal healthcare system. Lancet Reg Health West Pac.

[3] Williams J.W., Mulrow C.D., Kroenke K. (1999). Case-finding for depression in primary care: a randomized trial. Am J Med.

[4] Leung S.S.L., Leung C., Lam T.H. (2011). Outcome of a postnatal depression screening programme using the edinburgh postnatal depression scale: a randomized controlled trial. J Public Health.

[5] O'Connor E.A., Perdue L.A., Coppola E.L., Henninger M.L., Thomas R.G., Gaynes B.N. (2023). Depression and suicide risk screening: updated evidence report and systematic review for the US preventive services task force. JAMA.

[6] Reynolds C.F., Patel V. (2017). Screening for depression: the global mental health context. World Psychiatry.

[7] National UK Screening Committee (2018).

[8] Joffres M., Jaramillo A., Dickinson J. (2013). Recommendations on screening for depression in adults. CMAJ.

[9] Thombs B.D., Ziegelstein R.C., Roseman M., Kloda L.A., Ioannidis J.P.A. (2014). There are no randomized controlled trials that support the United States preventive services task force guideline on screening for depression in primary care: a systematic review. BMC Med.

[10] Thombs B.D., Saadat N., Riehm K.E. (2017). Consistency and sources of divergence in recommendations on screening with questionnaires for presently experienced health problems or symptoms: a comparison of recommendations from the Canadian task force on preventive health care, UK national screening. BMC Med.

[11] Hernán M.A., Robins J.M. (2016). Using big data to emulate a target trial when a randomized trial is not available. Am J Epidemiol.

[12] Whooley M.A., Avins A.L., Miranda J., Browner W.S. (1997). Case-finding instruments for depression: two questions are as good as many. J Gen Intern Med.

[13] Cheng A.T. (1995). Mental illness and suicide: a case-control study in East Taiwan. Arch Gen Psychiatry.

[14] Fine J.P., Gray R.J. (1999). A proportional hazards model for the subdistribution of a competing risk. J Am Stat Assoc.

[15] Whooley M.A., Stone B., Soghikian K. (2000). Randomized trial of case-finding for depression in elderly primary care patients. J Gen Intern Med.

[16] Romera I., Montejo Á.L., Aragonés E. (2013). Systematic depression screening in high-risk patients attending primary care: a pragmatic cluster-randomized trial. BMC Psychiatry.

[17] Miller E.S., Wisner K.L., Gollan J., Hamade S., Gossett D.R., Grobman W.A. (2019). Screening and treatment after implementation of a universal perinatal depression screening program. Obstet Gynecol.

[18] Premji S., McDonald S.W., Metcalfe A. (2019). Examining postpartum depression screening effectiveness in well child clinics in Alberta, Canada: a study using the all our families cohort and administrative data. Prev Med Rep.

[19] Burton C., Simpson C., Anderson N. (2013). Diagnosis and treatment of depression following routine screening in patients with coronary heart disease or diabetes: a database cohort study. Psychol Med.

[20] Pfoh E.R., Janmey I., Anand A. (2020). The impact of systematic depression screening in primary care on depression identification and treatment in a large health care system: a cohort study. J Gen Intern Med.

[21] Mackenzie C.S., Visperas A., Ogrodniczuk J.S., Oliffe J.L., Nurmi M.A. (2019). Age and sex differences in self-stigma and public stigma concerning depression and suicide in men. Stigma Health.

[22] Krivoy A., Balicer R.D., Feldman B. (2015). The impact of age and gender on adherence to antidepressants: a 4-year population-based cohort study. Psychopharmacology (Berl).

[23] Tunvirachaisakul C., Gould R.L., Coulson M.C. (2018). Predictors of treatment outcome in depression in later life: a systematic review and meta-analysis. J Affect Disord.

[24] Clement S., Schauman O., Graham T. (2015). What is the impact of mental health-related stigma on help-seeking? A systematic review of quantitative and qualitative studies. Psychol Med.

[25] Pompili M. (2019). Critical appraisal of major depression with suicidal ideation. Ann Gen Psychiatry.

[26] Bolton J.M., Walld R., Chateau D., Finlayson G., Sareen J. (2015). Risk of suicide and suicide attempts associated with physical disorders: a population-based, balancing score-matched analysis. Psychol Med.

[27] Rost K., Nutting P., Smith J., Werner J., Duan N. (2001). Improving depression outcomes in community primary care practice: a randomized trial of the quEST intervention. Quality enhancement by strategic teaming. J Gen Intern Med.

