# Supplementary material for: Effectiveness of health checkup with depression screening on depression treatment and outcomes in middle-aged and older adults: a target trial emulation study
Source: Lancet Reg Health West Pac. 2023 Nov 23;43:100978. doi: 10.1016/j.lanwpc.2023.100978 (PMC10701157; doi:10.1016/j.lanwpc.2023.100978)
Supplement: Supplementary Materials [file mmc1.docx]

Supplementary Table 1. **Protocol components of the hypothetical randomised trial and the emulation of a target trial for the effect of health checks with depression screening on mental health treatment and outcomes**

| **Protocol Component** | **Hypothetical Randomised Trial** | **Target Trial Emulation** |
| --- | --- | --- |
| **Eligibility criteria** | Inclusion criteria   1. aged 40 or above   Exclusion criteria   1. previously received depression screening 2. previously treated with depressive or bipolar-spectrum disorders in the past | Same, but limited to those who participated in Taiwan’s National Health Insurance program and had complete demographic variables in birth year, sex, monthly income, or residential area. |
| **Treatment strategies** | (1) depression screening  (2) no screening | Same |
| **Assignment procedures** | Participants are randomised to a treatment strategy at baseline and unaware of the assigned strategy. | Randomisation was assumed conditional on baseline covariates, including a residential area, incomes, number of medical conditions (Charlson comorbid index), number of outpatient visits, and medical and psychiatric disorders. |
| **Follow-up period** | Follow-up begins at time zero,  when an eligible individual is randomly assigned to one of the treatment strategies. Follow-up ends at the earliest of death, loss of follow-up, outcome occurrence, or study end. | Time zero was defined as “time participated in the health checkup with depression screening” for the screened individuals. For each screened individual on their entry date, a matched individual who was not screened was included in the control group. Follow-up ends at the earliest of death, loss of follow-up, outcome occurrence, or study end. |
| **Outcomes** | Newly-treated depression, psychiatric hospitalisation, and suicide | Newly-treated depression, psychiatric hospitalisation, and suicide |
| **Causal contrasts of interest** | Intention-to-treat and per-protocol effect | Intention-to-treat and per-protocol effect |
| **Analysis plan** | Competing risk survival analysis to estimate the hazard ratio (HR) and 95% confidence interval (CI) for study outcomes. Death is a competing risk. Censoring occurred for deviation from assigned strategies. | Same |

Supplementary Table 2. **Baseline characteristics of the study subjects**

|  | **Original** |  |  | **Propensity score weighting** |  |  |
| --- | --- | --- | --- | --- | --- | --- |
|  | **Depression Screening** | **No screening** | **SMD** | **Depression Screening** | **No screening** | **SMD** |
|  | **N (%)** | **N (%)** |  | **N (%)** | **N (%)** |  |
| **Age** |  |  |  |  |  |  |
| **40–54** | 2,231,921 (47%) | 2,231,921 (47%) | 0·00 | 2,241,025 (47%) | 2,210,131 (46%) | 0·01 |
| **55–64** | 1,426,794 (30%) | 1,426,794 (30%) | 0·00 | 1,416,217 (30%) | 1,446,745 (30%) | -0·01 |
| **≥ 65** | 1,133,513 (24%) | 1,133,513 (24%) | 0·00 | 1,134,986 (24%) | 1,135,352 (24%) | 0·00 |
| **Sex** |  |  |  |  |  |  |
| **Male** | 2,156,427 (45%) | 2,156,427 (45%) | 0·00 | 2,156,503 (45%) | 2,155,544 (45%) | 0·00 |
| **Female** | 2,635,801 (55%) | 2,635,801 (55%) | 0·00 | 2,635,725 (55%) | 2,636,684 (55%) | 0·00 |
| **Residency** |  |  |  |  |  |  |
| **Urban** | 2,299,180 (48%) | 2,639,768 (55%) | -0·10 | 2,469,914 (52%) | 2,469,682 (52%) | 0·00 |
| **Suburban** | 1,848,950 (39%) | 1,637,062 (34%) | 0·07 | 1,742,933 (36%) | 1,743,108 (36%) | 0·00 |
| **Rural** | 644,098 (13%) | 515,398 (11%) | 0·06 | 579,380 (12%) | 579,438 (12%) | 0·00 |
| **Monthly Income, NT$** |  |  |  |  |  |  |
| **≤ 20000** | 1,022,889 (21%) | 1,207,793 (25%) | -0·06 | 1,110,949 (23%) | 1,110,838 (23%) | 0·00 |
| **20001–40000** | 2,652,459 (55%) | 2,274,738 (47%) | 0·11 | 2,467,286 (51%) | 2,467,997 (52%) | 0·00 |
| **≥ 40000** | 1,116,880 (23%) | 1,309,697 (27%) | -0·07 | 1,213,993 (25%) | 1,213,392 (25%) | 0·00 |
| **Charlson comorbid index** |  |  |  |  |  |  |
| **0** | 3,644,693 (76%) | 3,708,478 (77%) | -0·02 | 3,668,930 (77%) | 3,670,001 (77%) | 0·00 |
| **1** | 720,978 (15%) | 622,534 (13%) | 0·04 | 675,704 (14%) | 675,637 (14%) | 0·00 |
| **2** | 274,023 (6%) | 283,634 (6%) | -0·01 | 280,825 (6%) | 280,317 (6%) | 0·00 |
| **≥ 3** | 152,534 (3%) | 177,582 (4%) | -0·02 | 166,770 (3%) | 166,274 (3%) | 0·00 |
| **Number of outpatient visits** |  |  |  |  |  |  |
| **0–9** | 2,454,637 (51%) | 2,926,356 (61%) | -0·14 | 2,688,919 (56%) | 2,688,709 (56%) | 0·00 |
| **10–19** | 1,173,050 (24%) | 955,273 (20%) | 0·08 | 1,064,833 (22%) | 1,064,939 (22%) | 0·00 |
| **≥ 20** | 1,164,541 (24%) | 910,599 (19%) | 0·09 | 1,038,476 (22%) | 1,038,580 (22%) | 0·00 |
| **Calendar year at screening** |  |  |  |  |  |  |
| **2013** | 1,130,050 (24%) | 1,130,050 (24%) | 0·00 | 1,136,351 (24%) | 1,137,196 (24%) | 0·00 |
| **2014** | 985,693 (21%) | 985,693 (21%) | 0·00 | 997,362 (21%) | 996,783 (21%) | 0·00 |
| **2015** | 693,702 (14%) | 693,702 (14%) | 0·00 | 691,588 (14%) | 691,519 (14%) | 0·00 |
| **2016** | 554,303 (12%) | 554,303 (12%) | 0·00 | 545,889 (11%) | 545,835 (11%) | 0·00 |
| **2017** | 507,833 (11%) | 507,833 (11%) | 0·00 | 499,879 (10%) | 499,829 (10%) | 0·00 |
| **2018** | 465,516 (10%) | 465,516 (10%) | 0·00 | 458,183 (10%) | 458,137 (10%) | 0·00 |
| **2019** | 455,131 (10%) | 455,131 (10%) | 0·00 | 462,975 (10%) | 462,929 (10%) | 0·00 |
| **Comorbidity** |  |  |  |  |  |  |
| **Hypertension** | 1,124,525 (23%) | 978,840 (20%) | 0·05 | 1,058,603 (22%) | 1,058,603 (22%) | 0·00 |
| **Dyslipidaemia** | 611,999 (13%) | 589,138 (12%) | 0·01 | 606,217 (13%) | 605,258 (13%) | 0·00 |
| **Diabetes** | 417,306 (9%) | 448,086 (9%) | -0·02 | 437,051 (9%) | 435,614 (9%) | 0·00 |
| **Chronic liver disease** | 239,672 (5%) | 212,622 (4%) | 0·02 | 227,631 (5%) | 228,110 (5%) | 0·00 |
| **Chronic kidney disease** | 77,163 (2%) | 111,492 (2%) | -0·04 | 95,845 (2%) | 94,886 (2%) | 0·00 |
| **Chronic pulmonary disease** | 212,717 (4%) | 168,678 (4%) | 0·03 | 191,210 (4%) | 191,689 (4%) | 0·00 |
| **Cardiovascular disorder** | 78,317 (2%) | 89,323 (2%) | -0·01 | 84,343 (2%) | 83,864 (2%) | 0·00 |
| **Peptic ulcer** | 272,905 (6%) | 209,841 (4%) | 0·04 | 242,008 (5%) | 242,008 (5%) | 0·00 |
| **Cerebrovascular disorders** | 153,856 (3%) | 162,837 (3%) | -0·01 | 159,581 (3%) | 159,102 (3%) | 0·00 |
| **Peripheral vascular disorder** | 28,662 (1%) | 26,287 (1%) | 0·00 | 27,507 (1%) | 27,507 (1%) | 0·00 |
| **Rheumatic disorders** | 41,022 (1%) | 44,199 (1%) | 0·00 | 42,938 (1%) | 42,843 (1%) | 0·00 |
| **Dementia** | 38,781 (1%) | 35,898 (1%) | 0·00 | 37,619 (1%) | 37,619 (1%) | 0·00 |
| **Psychosomatic disorders** | 12,078 (0%) | 7,403 (0%) | 0·02 | 9,824 (0%) | 9,872 (0%) | 0·00 |
| **Sleep** | 308,542 (6%) | 231,195 (5%) | 0·05 | 270,761 (6%) | 271,240 (6%) | 0·00 |
| **Acute stress disorder/posttraumatic stress disorder** | 4,234 (0%) | 2,588 (0%) | 0·01 | 3,441 (0%) | 3,465 (0%) | 0·00 |
| **Organic brain disorder** | 10,209 (0%) | 9,453 (0%) | 0·00 | 9,920 (0%) | 9,920 (0%) | 0·00 |
| **Schizophrenia** | 22,834 (0%) | 22,243 (0%) | 0·00 | 22,571 (0%) | 22,571 (0%) | 0·00 |
| **Delusional disorder** | 1,695 (0%) | 1,537 (0%) | 0·00 | 1,620 (0%) | 1,615 (0%) | 0·00 |
| **Panic disorder** | 8,008 (0%) | 5,679 (0%) | 0·01 | 6,853 (0%) | 6,853 (0%) | 0·00 |
| **Generalised anxiety disorder** | 37,554 (1%) | 25,096 (1%) | 0·02 | 31,485 (1%) | 31,581 (1%) | 0·00 |
| **Phobic disorder and agoraphobia** | 1,069 (0%) | 879 (0%) | 0·00 | 968 (0%) | 968 (0%) | 0·00 |
| **Obsessive-compulsive disorder** | 1,453 (0%) | 1,350 (0%) | 0·00 | 1,404 (0%) | 1,404 (0%) | 0·00 |
| **Alcohol or substance use disorder** | 6,644 (0%) | 5,980 (0%) | 0·00 | 6,374 (0%) | 6,374 (0%) | 0·00 |

SMD: standardised mean difference; NT$: New Taiwan Dollar; average exchange rate in 2019: 30·8869 TWD for 1 US dollar

Supplementary Table 3. **Competing hazard ratios of depression screening for newly treated depression, psychiatric hospitalisation, and suicide from intention-to-treat analyses with propensity score weighting**

|  | **Depression screening** |  |  | **No screening** |  |  |  |  |
| --- | --- | --- | --- | --- | --- | --- | --- | --- |
|  | **No. of events** | **No. of 1,000 person-years** | **Rate per 1,000 person-years** | **No. of events** | **No. of 1,000 person-years** | **Rate per 1,000 person-years** | **Hazard ratios (95% CI)** | **p value** |
| **Newly-treated depression** |  |  |  |  |  |  |  |  |
| **Overall** | 200,817 | 19,155 | 10·48 | 144,430 | 19,199 | 7·52 | 1·42 (1·41, 1·43) | < ·001 |
| **Age groups** |  |  |  |  |  |  |  |  |
| **40–54** | 82,199 | 8,664 | 9.49 | 57,301 | 8,733 | 6.56 | 1.42 (1.41, 1.44) | < ·001 |
| **55–64** | 60,842 | 6,001 | 10.14 | 44,069 | 6,024 | 7.32 | 1.37 (1.35, 1.39) | < ·001 |
| **≥ 65** | 57,545 | 4,489 | 12.82 | 43,211 | 4,442 | 9.73 | 1.32 (1.30, 1.34) | < ·001 |
| **Sex** |  |  |  |  |  |  |  |  |
| **Male** | 70,134 | 8,442 | 8.31 | 48,706 | 8,446 | 5.77 | 1.42 (1.41, 1.44) | < ·001 |
| **Female** | 130,666 | 10,712 | 12.20 | 95,751 | 10,754 | 8.90 | 1.36 (1.35, 1.37) | < ·001 |
| **Urbanicity** |  |  |  |  |  |  |  |  |
| **Urban** | 96,102 | 9,027 | 10.65 | 78,766 | 10,410 | 7.57 | 1.39 (1.38, 1.40) | < ·001 |
| **Suburban** | 77,864 | 7,519 | 10.36 | 49,852 | 6,663 | 7.48 | 1.37 (1.36, 1.39) | < ·001 |
| **Rural** | 27,007 | 2,641 | 10.22 | 15,827 | 2,110 | 7.50 | 1.35 (1.33, 1.38) | < ·001 |
| **Psychiatric hospitalisation** |  |  |  |  |  |  |  |  |
| **Overall** | 15,158 | 19,699 | 0·77 | 15,723 | 19,558 | 0·80 | 0·96 (0·94, 0·98) | 0·001 |
| **Age groups** |  |  |  |  |  |  |  |  |
| **40–54** | 7,613 | 8,884 | 0.86 | 7,726 | 8,871 | 0.87 | 0.99 (0.96, 1.03) | 0.702 |
| **55–64** | 4,063 | 6,172 | 0.66 | 4,667 | 6,138 | 0.76 | 0.88 (0.84, 0.91) | <.001 |
| **≥ 65** | 3,416 | 4,641 | 0.74 | 3,390 | 4,549 | 0.75 | 1.01 (0.96, 1.06) | 0.773 |
| **Sex** |  |  |  |  |  |  |  |  |
| **Male** | 7,983 | 8,616 | 0.93 | 7,851 | 8,554 | 0.92 | 1.03 (0.99, 1.06) | 0.114 |
| **Female** | 7,165 | 11,082 | 0.65 | 7,907 | 11,006 | 0.72 | 0.91 (0.88, 0.94) | <.001 |
| **Urbanicity** |  |  |  |  |  |  |  |  |
| **Urban** | 6,867 | 9,285 | 0.74 | 7,994 | 10,607 | 0.75 | 0.99 (0.96, 1.02) | 0.590 |
| **Suburban** | 5,833 | 7,733 | 0.75 | 5,361 | 6,788 | 0.79 | 0.97 (0.93, 1.00) | 0.086 |
| **Rural** | 2,548 | 2,712 | 0.94 | 2,234 | 2,148 | 1.04 | 0.92 (0.87, 0.98) | 0.005 |
| **Suicide** |  |  |  |  |  |  |  |  |
| **Overall** | 3,414 | 19,741 | 0·17 | 3,343 | 19,602 | 0·17 | 1·02 (0·97, 1·07) | 0·429 |
| **Age groups** |  |  |  |  |  |  |  |  |
| **40–54** | 1,153 | 8,906 | 0.13 | 1,184 | 8,893 | 0.13 | 0.97 (0.90, 1.06) | 0.517 |
| **55–64** | 982 | 6,183 | 0.16 | 974 | 6,151 | 0.16 | 1.01 (0.92, 1.10) | 0.882 |
| **≥ 65** | 1,276 | 4,650 | 0.27 | 1,183 | 4,558 | 0.26 | 1.07 (0.99, 1.16) | 0.079 |
| **Sex** |  |  |  |  |  |  |  |  |
| **Male** | 2,319 | 8,638 | 0.27 | 2,289 | 8,575 | 0.27 | 1.01 (0.95, 1.07) | 0.708 |
| **Female** | 1,101 | 11,102 | 0.10 | 1,045 | 11,029 | 0.09 | 1.05 (0.97, 1.14) | 0.245 |
| **Urbanicity** |  |  |  |  |  |  |  |  |
| **Urban** | 1,370 | 9,305 | 0.15 | 1,600 | 10,629 | 0.15 | 0.98 (0.91, 1.06) | 0.646 |
| **Suburban** | 1,437 | 7,750 | 0.19 | 1,224 | 6,803 | 0.18 | 1.04 (0.96, 1.12) | 0.358 |
| **Rural** | 651 | 2,719 | 0.24 | 487 | 2,154 | 0.23 | 1.06 (0.95, 1.20) | 0.299 |

Supplementary Table 4. Competing hazard ratios of depression screening for newly treated depression, psychiatric hospitalisation, and suicide using unweighted data with multivariate Cox regression model

|  | **Depression screening** |  |  | **No screening** |  |  |  |  |  |  |
| --- | --- | --- | --- | --- | --- | --- | --- | --- | --- | --- |
|  | **No of events** | **No of 1,000 person-years** | **Rate per 1,000 person-years** | **No of events** | **No of 1,000 person-years** | **Rate per 1,000 person-years** | **Crude Hazard ratios (99% CI)** | **p-value** | **Adjusted Hazard ratios (99% CI)** | **p-value** |
| **Newly treated depression** |  |  |  |  |  |  |  |  |  |  |
| **Overall** | 206,950 | 19,101 | 10·83 | 96,058 | 15,188 | 6·32 | 1·78 (1·76, 1·79) | <·001 | 1·63 (1·62, 1·64) | <·001 |
| **Age groups** |  |  |  |  |  |  |  |  |  |  |
| **40-54** | 85,882 | 8,642 | 9·94 | 39,093 | 7,186 | 5·44 | 1·84 (1·82, 1·86) | <·001 | 1·63 (1·61, 1·65) | <·001 |
| **55-64** | 62,461 | 5,984 | 10·44 | 27,934 | 4,585 | 6·09 | 1·76 (1·73, 1·78) | <·001 | 1·62 (1·59, 1·64) | <·001 |
| **≥ 65** | 58,607 | 4,475 | 13·10 | 29,031 | 3,417 | 8·50 | 1·61 (1·59, 1·63) | <·001 | 1·54 (1·52, 1·56) | <·001 |
| **Sex** |  |  |  |  |  |  |  |  |  |  |
| **Male** | 72,254 | 8,415 | 8·59 | 34,010 | 6,922 | 4·91 | 1·77 (1·75, 1·80) | <·001 | 1·64 (1·62, 1·66) | <·001 |
| **Female** | 134,696 | 10,686 | 12·60 | 62,048 | 8,267 | 7·51 | 1·72 (1·70, 1·74) | <·001 | 1·59 (1·57, 1·60) | <·001 |
| **Urbanicity** |  |  |  |  |  |  |  |  |  |  |
| **Urban** | 98,807 | 8,947 | 11·04 | 55,227 | 8,517 | 6·48 | 1·73 (1·71, 1·75) | <·001 | 1·58 (1·57, 1·60) | <·001 |
| **Suburban** | 80,222 | 7,505 | 10·69 | 31,316 | 5,108 | 6·13 | 1·79 (1·77, 1·82) | <·001 | 1·63 (1·61, 1·66) | <·001 |
| **Rural** | 27,921 | 2,649 | 10·54 | 9,515 | 1,563 | 6·06 | 1·79 (1·75, 1·83) | <·001 | 1·63 (1·59, 1·67) | <·001 |
| **Psychiatric hospitalisation** |  |  |  |  |  |  |  |  |  |  |
| **Overall** | 15,124 | 19,662 | 0·77 | 13,625 | 15,436 | 0·88 | 0·93 (0·91, 0·95) | <·001 | 0·91 (0·89, 0·94) | <·001 |
| **Age groups** |  |  |  |  |  |  |  |  |  |  |
| **40-54** | 7,907 | 8,871 | 0·89 | 6,583 | 7,284 | 0·90 | 1·04 (1·01, 1·08) | 0·013 | 0·95 (0·91, 0·98) | 0·002 |
| **55-64** | 3,886 | 6,161 | 0·63 | 4,258 | 4,660 | 0·91 | 0·74 (0·71, 0·77) | <·001 | 0·80 (0·77, 0·84) | <·001 |
| **≥ 65** | 3,331 | 4,630 | 0·72 | 2,784 | 3,493 | 0·80 | 0·97 (0·92, 1·02) | 0·224 | 0·97 (0·92, 1·03) | 0·344 |
| **Sex** |  |  |  |  |  |  |  |  |  |  |
| **Male** | 8,170 | 8,595 | 0·95 | 6,669 | 6,999 | 0·95 | 1·06 (1·03, 1·10) | <·001 | 0·97 (0·94, 1·01) | 0·133 |
| **Female** | 6,954 | 11,067 | 0·63 | 6,956 | 8,437 | 0·82 | 0·81 (0·78, 0·84) | <·001 | 0·86 (0·83, 0·89) | <·001 |
| **Urbanicity** |  |  |  |  |  |  |  |  |  |  |
| **Urban** | 6,901 | 9,213 | 0·75 | 6,938 | 8,661 | 0·80 | 0·98 (0·95, 1·02) | 0·287 | 0·94 (0·91, 0·98) | 0·001 |
| **Suburban** | 5,738 | 7,727 | 0·74 | 4,703 | 5,189 | 0·91 | 0·88 (0·85, 0·92) | <·001 | 0·91 (0·87, 0·95) | <·001 |
| **Rural** | 2,485 | 2,723 | 0·91 | 1,984 | 1,586 | 1·25 | 0·81 (0·76, 0·86) | <·001 | 0·86 (0·80, 0·91) | <·001 |
| **Suicide** |  |  |  |  |  |  |  |  |  |  |
| **Overall** | 3,514 | 19,704 | 0·18 | 2,587 | 15,472 | 0·17 | 1·07(1·01,1·12) | 0·015 | 1·01(0·96,1·06) | 0·676 |
| **Age groups** |  |  |  |  |  |  |  |  |  |  |
| **40-54** | 1,205 | 8,894 | 0·14 | 991 | 7,302 | 0·14 | 0·98(0·91,1·07) | 0·720 | 0·93(0·85,1·01) | 0·075 |
| **55-64** | 996 | 6,171 | 0·16 | 771 | 4,671 | 0·17 | 0·98(0·89,1·07) | 0·610 | 0·96(0·87,1·05) | 0·388 |
| **≥ 65** | 1,313 | 4,638 | 0·28 | 825 | 3,499 | 0·24 | 1·24(1·13,1·35) | <·001 | 1·15(1·06,1·26) | 0·002 |
| **Sex** |  |  |  |  |  |  |  |  |  |  |
| **Male** | 2,382 | 8,617 | 0·28 | 1,811 | 7,016 | 0·26 | 1·07(1·01,1·14) | 0·023 | 1·01(0·95,1·07) | 0·806 |
| **Female** | 1,132 | 11,087 | 0·10 | 776 | 8,456 | 0·09 | 1·10(1·00,1·20) | 0·047 | 1·03(0·94,1·13) | 0·481 |
| **Urbanicity** |  |  |  |  |  |  |  |  |  |  |
| **Urban** | 1,388 | 9,232 | 0·15 | 1,323 | 8,679 | 0·15 | 0·98(0·91,1·06) | 0·678 | 0·96(0·89,1·03) | 0·251 |
| **Suburban** | 1,462 | 7,743 | 0·19 | 907 | 5,202 | 0·17 | 1·09(1·00,1·18) | 0·047 | 1·06(0·97,1·15) | 0·198 |
| **Rural** | 664 | 2,729 | 0·24 | 357 | 1,591 | 0·22 | 1·08(0·95,1·23) | 0·225 | 1·05(0·92,1·20) | 0·478 |

Supplementary Table 5. **Interval Cox regression model**

|  | **Depression screening** |  |  | **No screening** |  |  |  |  |  |  |
| --- | --- | --- | --- | --- | --- | --- | --- | --- | --- | --- |
|  | **No. of events** | **No. of 1,000 person-years** | **Rate per 1,000 person-years** | **No. of events** | **No. of 1,000 person-years** | **Rate per 1,000 person-years** | **Crude Hazard ratios (99% CI)** | **p value** | **Adjusted Hazard ratios (99% CI)** | **p value** |
| **Newly-treated Depression** |  |  |  |  |  |  |  |  |  |  |
| **≤ 90** | 23,935 | 1,170 | 20·46 | 8,366 | 1,161 | 7·21 | 2·49 (2·43,2·56) | < ·001 | 2·29 (2·23, 2·34) | < ·001 |
| **91–180** | 14,668 | 1,140 | 12·86 | 7,827 | 1,102 | 7·10 | 1·76 (1·71,1·81) | < ·001 | 1·60 (1·56,1·65) | < ·001 |
| **181–365** | 25,477 | 2,232 | 11·41 | 14,172 | 2,076 | 6·83 | 1·66 (1·63,1·69) | < ·001 | 1·50 (1·47,1·54) | < ·001 |
| **1y–3y** | 76,974 | 7,461 | 10·32 | 38,395 | 6,164 | 6·23 | 1·64 (1·62,1·66) | < ·001 | 1·50 (1·48,1·52) | < ·001 |
| **3y–7y** | 65,896 | 7,281 | 9·05 | 25,870 | 4,818 | 5·37 | 1·68 (1·66,1·71) | < ·001 | 1·56 (1·53,1·58) | < ·001 |
| **Psychiatric hospitalisation** |  |  |  |  |  |  |  |  |  |  |
| **≤ 90** | 1,746 | 1,173 | 1·49 | 1,796 | 1,162 | 1·55 | 1·07 (1·00,1·14) | 0·053 | 1·07 (1·00,1·15) | 0·039 |
| **91–180** | 1,448 | 1,147 | 1·26 | 1,408 | 1,105 | 1·27 | 0·90 (0·84,0·97) | 0·008 | 0·89 (0·83,0·96) | 0·002 |
| **181–365** | 2,176 | 2,255 | 0·96 | 2,321 | 2,085 | 1·11 | 0·89 (0·84,0·95) | 0·000 | 0·87 (0·82,0·93) | < ·001 |
| **1y–3y** | 5,426 | 7,633 | 0·71 | 4,946 | 6,242 | 0·79 | 0·91 (0·87,0·94) | < ·001 | 0·88 (0·85,0·92) | < ·001 |
| **3y–7y** | 4,328 | 7,637 | 0·57 | 3,133 | 4,975 | 0·63 | 0·91 (0·87,0·95) | < ·001 | 0·89 (0·85,0·93) | < ·001 |
| **Suicide** |  |  |  |  |  |  |  |  |  |  |
| **≤ 90** | 160 | 1,173 | 0·14 | 114 | 1,162 | 0·10 | 1·39 (1·12,1·72) | 0·003 | 1·30 (1·04,1·61) | 0·021 |
| **91–180** | 214 | 1,148 | 0·19 | 160 | 1,105 | 0·14 | 1·10 (0·90,1·35) | 0·357 | 1·04 (0·85,1·27) | 0·693 |
| **181–365** | 396 | 2,257 | 0·18 | 344 | 2,087 | 0·16 | 1·11 (0·96,1·29) | 0·146 | 1·03 (0·89,1·20) | 0·658 |
| **1y–3y** | 1,313 | 7,647 | 0·17 | 1,030 | 6,255 | 0·16 | 1·05 (0·97,1·14) | 0·239 | 0·99 (0·91,1·08) | 0·809 |
| **3y–7y** | 1,431 | 7,662 | 0·19 | 939 | 4,995 | 0·19 | 1·00 (0·92,1·09) | 0·978 | 0·95 (0·87,1·03) | 0·218 |

Supplementary Table 6. **Baseline characteristics of patients with newly treated depressive disorders stratified by screening status.**

|  | **Unscreened** | **Screened (-)** | **Screened (+)** |
| --- | --- | --- | --- |
| **Age groups** |  |  |  |
| **Mean ± SD** | 60·79 ± 12·19 | 60·53 ± 11·92 | 58·74 ± 11·71 |
| **40–54** | 33,340 (35%) | 66,773 (35%) | 5,503 (41%) |
| **55–64** | 28,348 (30%) | 58,718 (30%) | 4,088 (30%) |
| **≥ 65** | 34,370 (35%) | 67,901 (35%) | 3,968 (29%) |
| **Sex** |  |  |  |
| **Male** | 34,010 (35%) | 67,588 (35%) | 4,667 (34%) |
| **Female** | 62,048 (65%) | 125,804 (65%) | 8,892 (66%) |
| **Residency** |  |  |  |
| **Urban** | 55,227 (57%) | 91,352 (47%) | 7,456 (55%) |
| **Suburban** | 31,316 (33%) | 75,620 (39%) | 4,602 (34%) |
| **Rural** | 9,515 (10%) | 26,420 (14%) | 1,501 (11%) |
| **Monthly Income, NT$** |  |  |  |
| **≤20000** | 28,434 (30%) | 49,569 (26%) | 4,056 (30%) |
| **20001–40000** | 44,141 (46%) | 105,752 (55%) | 6,802 (50%) |
| **≥ 40000** | 23,483 (24%) | 38,071 (20%) | 2,701 (20%) |
| **Charlson comorbid index** |  |  |  |
| **0** | 52,649 (55%) | 99,929 (52%) | 7,618 (56%) |
| **1** | 20,732 (22%) | 49,876 (26%) | 3,344 (25%) |
| **2** | 11,210 (12%) | 23,532 (12%) | 1,454 (11%) |
| **≥ 3** | 11,467 (12%) | 20,055 (10%) | 1,143 (8%) |
| **Number of outpatient visits** |  |  |  |
| **0–9** | 23,408 (24%) | 24,270 (13%) | 2,394 (18%) |
| **10–19** | 22,878 (24%) | 42,124 (22%) | 3,165 (23%) |
| **≥ 20** | 49,772 (52%) | 126,998 (66%) | 8,000 (59%) |
| **Calendar year** |  |  |  |
| **2013** | 6,601 (7%) | 11,385 (6%) | 1,545 (11%) |
| **2014** | 13,039 (14%) | 23,075 (12%) | 2,146 (16%) |
| **2015** | 16,331 (17%) | 29,835 (15%) | 2,254 (17%) |
| **2016** | 15,806 (16%) | 30,398 (16%) | 2,010 (15%) |
| **2017** | 15,144 (16%) | 32,202 (17%) | 1,967 (15%) |
| **2018** | 14,924 (16%) | 33,269 (17%) | 1,850 (14%) |
| **2019** | 14,213 (15%) | 33,228 (17%) | 1,787 (13%) |
| **Comorbidity** |  |  |  |
| **Hypertension** | 34,633 (36%) | 75,830 (39%) | 4,429 (33%) |
| **Dyslipidemia** | 20,576 (21%) | 50,974 (26%) | 2,932 (22%) |
| **Diabetes** | 14,405 (15%) | 24,839 (13%) | 1,447 (11%) |
| **Chronic liver disease** | 8,258 (9%) | 21,042 (11%) | 1,355 (10%) |
| **Chronic kidney disease** | 5,931 (6%) | 9,331 (5%) | 491 (4%) |
| **Chronic pulmonary disease** | 8,175 (9%) | 18,895 (10%) | 1,273 (9%) |
| **Cardiovascular disorder** | 4,356 (5%) | 7,736 (4%) | 411 (3%) |
| **Peptic ulcer** | 12,019 (13%) | 33,771 (17%) | 2,203 (16%) |
| **Cerebrovascular disorders** | 9,805 (10%) | 17,288 (9%) | 1,098 (8%) |
| **Peripheral vascular disorder** | 1,201 (1%) | 2,649 (1%) | 139 (1%) |
| **Rheumatic disorders** | 1,890 (2%) | 3,633 (2%) | 263 (2%) |
| **Dementia** | 4,439 (5%) | 7,393 (4%) | 478 (4%) |
| **Psychosomatic disorder** | 806 (1%) | 2,571 (1%) | 187 (1%) |
| **Sleep** | 26,273 (27%) | 65,306 (34%) | 4,642 (34%) |
| **Acute stress disorder/posttraumatic stress disorder** | 379 (0%) | 1,149 (1%) | 72 (1%) |
| **Organic brain disorder** | 1,068 (1%) | 1,930 (1%) | 152 (1%) |
| **Schizophrenia** | 1,386 (1%) | 2,183 (1%) | 216 (2%) |
| **Delusional disorder** | 284 (0%) | 413 (0%) | 41 (0%) |
| **Panic disorder** | 1,397 (1%) | 3,260 (2%) | 314 (2%) |
| **Generalised anxiety disorder** | 5,593 (6%) | 15,652 (8%) | 1,098 (8%) |
| **Phobic disorder and agoraphobia** | 187 (0%) | 420 (0%) | 38 (0%) |
| **Obsessive-compulsive disorder** | 268 (0%) | 447 (0%) | 47 (0%) |
| **Alcohol or substance use disorder** | 1,118 (1%) | 1,863 (1%) | 166 (1%) |

NT$: New Taiwan Dollar; average exchange rate in 2019: 30·8869 TWD for 1 US dollar

**Supplementary Table 7. Post hoc analysis for the risk of psychiatric hospitalization and suicide death among patients with newly treated depression using multivariate Cox regression models by screening status**

|  | **No. of subjects** | **No. of events** | **No. of 1,000 person-years** | **Rate per 1,000 person-years** | **Crude Hazard ratios (95% CI)** | **p value** | **Adjusted Hazard ratios (95% CI)** | **p value** |
| --- | --- | --- | --- | --- | --- | --- | --- | --- |
| **Psychiatric hospitalisation** |  |  |  |  |  |  |  |  |
| **No-screening** | 96,058 | 2,069 | 278 | 7.4 |  |  |  |  |
| **Screen-negative** | 193,391 | 3,435 | 549 | 6.3 | 0.83 (0.79, 0.88) | <.001 | 0.88 (0.84, 0.93) | <.001 |
| **Screen-positive** | 13,559 | 295 | 45 | 6.6 | 0.96 (0.85,1.09) | 0.546 | 0.94 (0.84, 1.07) | 0.360 |
| **Suicidal death** |  |  |  |  |  |  |  |  |
| **No-screening** | 96,058 | 424 | 283 | 1.5 |  |  |  |  |
| **Screen-negative** | 193,391 | 855 | 557 | 1.5 | 1.03 (0.91, 1.15) | 0.671 | 0.97 (0.86, 1.10) | 0.656 |
| **Screen-positive** | 13,559 | 60 | 45 | 1.3 | 0.94 (0.72, 1.23) | 0.637 | 0.88 (0.67, 1.16) | 0.365 |
