# Supplementary figures and images for: Effectiveness of health checkup with depression screening on depression treatment and outcomes in middle-aged and older adults: a target trial emulation study
Source: Lancet Reg Health West Pac. 2023 Nov 23;43:100978. doi: 10.1016/j.lanwpc.2023.100978 (PMC10701157; doi:10.1016/j.lanwpc.2023.100978)

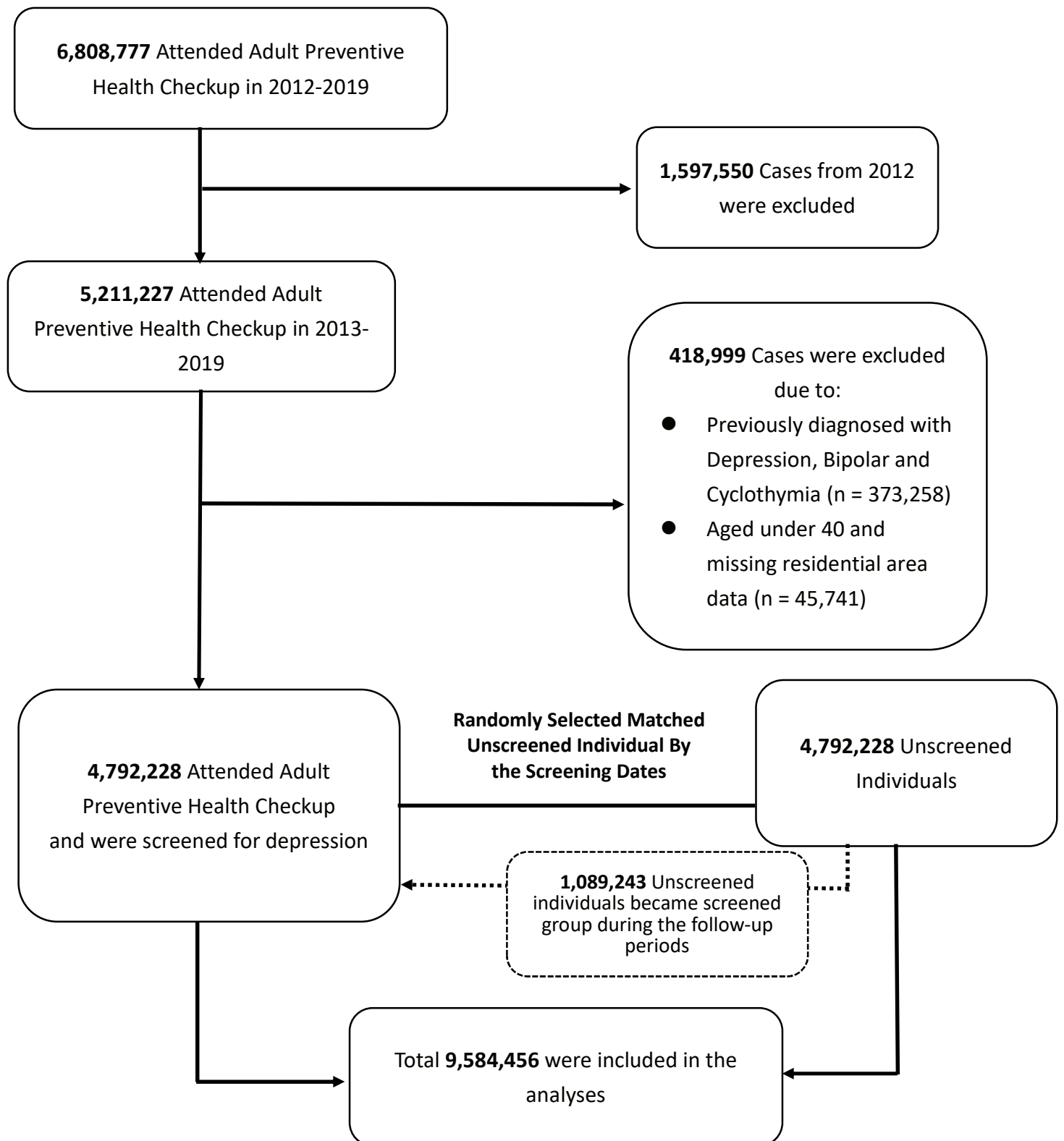

Supplement: Supplementary file 2 — Supplementary Figure S1 [file mmc2.pdf]

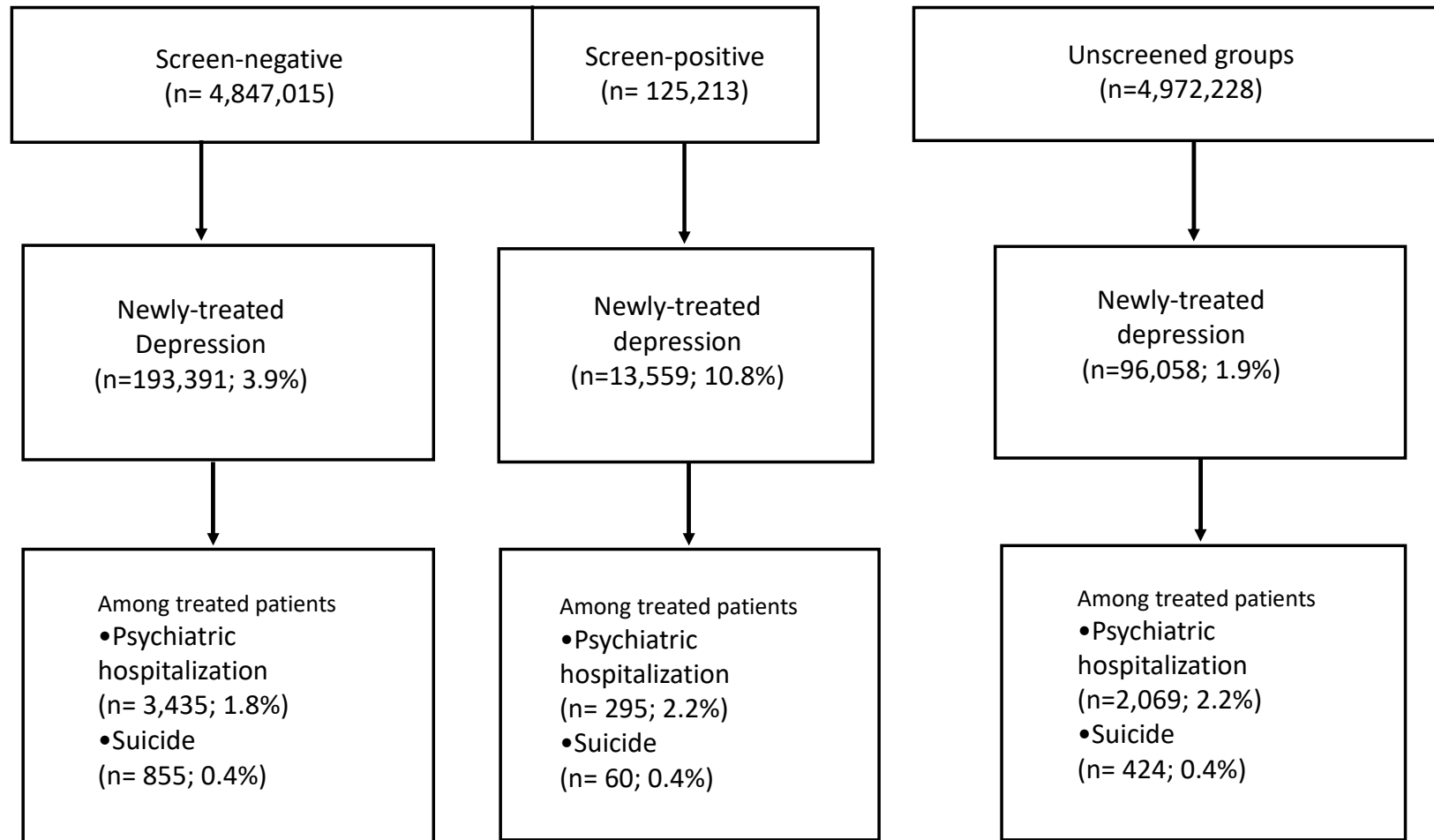

Supplement: Supplementary Figure S3 [file mmc3.pdf]
